# Insight into the Latest Medical Applications of Nanocellulose

**DOI:** 10.3390/ma16124447

**Published:** 2023-06-17

**Authors:** Alina Ghilan, Raluca Nicu, Diana E. Ciolacu, Florin Ciolacu

**Affiliations:** 1 Department of Natural Polymers, Bioactive and Biocompatible Materials, “Petru Poni” Institute of Macromolecular Chemistry, 700487 Iasi, Romania; diaconu.alina@icmpp.ro (A.G.); nicu.raluca@icmpp.ro (R.N.); 2 Department of Natural and Synthetic Polymers, “Gheorghe Asachi” Technical University of Iasi, 700050 Iasi, Romania

**Keywords:** nanocellulose, biological properties, tissue engineering, drug delivery, wound dressing

## Abstract

Nanocelluloses (NCs) are appealing nanomaterials that have experienced rapid development in recent years, with great potential in the biomedical field. This trend aligns with the increasing demand for sustainable materials, which will contribute both to an improvement in wellbeing and an extension of human life, and with the demand to keep up with advances in medical technology. In recent years, due to the diversity of their physical and biological properties and the possibility of tuning them according to the desired goal, these nanomaterials represent a point of maximum interest in the medical field. Applications such as tissue engineering, drug delivery, wound dressing, medical implants or those in cardiovascular health are some of the applications in which NCs have been successfully used. This review presents insight into the latest medical applications of NCs, in the forms of cellulose nanocrystals (CNCs), cellulose nanofibers (CNFs) and bacterial nanocellulose (BNC), with an emphasis on the domains that have recently experienced remarkable growth, namely wound dressing, tissue engineering and drug delivery. In order to highlight only the most recent achievements, the presented information is focused on studies from the last 3 years. Approaches to the preparation of NCs are discussed either by top-down (chemical or mechanical degradation) or by bottom-up (biosynthesis) techniques, along with their morphological characterization and unique properties, such as mechanical and biological properties. Finally, the main challenges, limitations and future research directions of NCs are identified in a sustained effort to identify their effective use in biomedical fields.

## 1. Introduction

Nanocelluloses (NCs) represent a unique category of engineering materials made from cellulose and are considered one of the most promising green resources of today due to their particular properties [1]. NCs are attractive for a wide range of applications, including biomedical engineering (e.g., medical implants, wound healing, skin sensors) [2], smart packaging materials [3], environmental remediation (e.g., water and air filtration membranes, photocatalysis, flocculation) [4] or energy harvesting and storage (e.g., fuel cell membranes, stretchable and flexible electrodes) [5]. Widespread interest in NCs-based materials in medical applications has been centered on their availability, sustainability, low cost, biodegradability, biocompatibility, outstanding mechanical properties and low cytotoxicity [6].

Over the last ten years, the number of scientific publications related to NCs has increased significantly, but the most explosive growth was recorded in the last three years, namely between 2020 and 2022, when more than 50% of the publications were published. Regarding the term “nanocellulose”, searches in the Web of Science (WOS) online database core collection for the period 2020–2022 led to the finding of a total of 5018 publications, and of these, 3876 publications (i.e., more than 77%) were found with the topic keywords “tissue engineering”, “drug delivery” and “wound dressing”, respectively.

**Figure 1:**
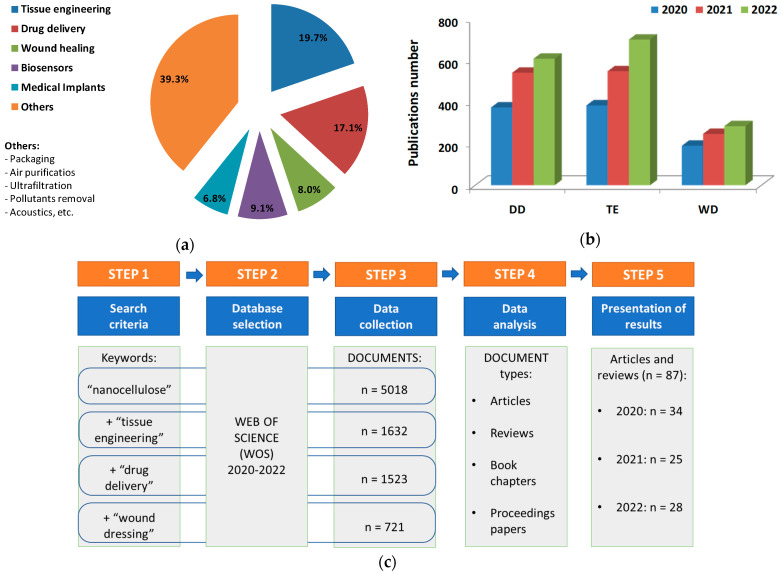
(**a**) The proportion of nanocellulose applications included in scientific publications from 2022 (Web of Science databases on March 2023, using the search terms “nanocellulose”); (**b**) the number of scientific publications from 2020 to 2022, using the topic keywords “drug delivery” (DD), “tissue engineering” (TE) and “wound dressing” (WD), respectively, in the Web of Science databases on March 2023; (**c**) step-by-step analysis for identifying and selecting the documents.

Starting from the abundance of articles related to NCs, we wanted to highlight the contribution of scientific publications for each type of medical application, precisely to emphasize the importance of the use of NCs in these fields in the last three years. Thus, in Figure 1a, it can be observed that for the year 2022, from all the fields in which NCs are used, more than 60% are represented by the medical field, and more precisely by tissue engineering (TE), drug delivery (DD), wound dressing (WD), biosensors and medical implants. Of the other applications, approximately 40% of the total are represented by domains such as packaging, air purification, ultrafiltration, pollutants removal, acoustics, etc.

Moreover, in the last three years, the number of the scientific publications regarding the applications of nanocellulose in the TE and DD fields recorded an increase of approximately 80% and 60%, respectively, as can be seen in Figure 1b. The applications of NCs-based materials in WD have also seen a significant increase of approximately 40% during the same period.

The step-by-step analysis for the identification and selection of the documents that were used in writing this review is schematically presented in Figure 1c. This analysis consists of several predetermined steps: (Step 1) Search criteria—the keywords necessary for the preparation of this review were selected, these being “nanocellulose”, “tissue engineering”, “drug delivery” and “ wound dressing”; (Step 2) selection of the database—a scientific, relevant and multidisciplinary database was chosen, this being the Web of Science (WOS) database; (Step 3) data collection—documents published in the period 2020–2022 were searched using different keywords, the final result consisting of a number of representative documents for the respective field. For “nanocellulose,” 5018 documents were obtained, while for the keyword combinations “nanocellulose + tissue engineering,” there was 1632, for “nanocellulose + drug delivery”, there was 1523, and for “nanocellulose + wound dressing”, there was 721; (Step 4) data analysis—after data collection, we proceeded to (i) identify the selected documents when it was noticed that they contained articles, reviewers, book chapters, proceedings papers, etc., (ii) evaluate the documents while taking into account their relevance, publication year, impact factor and analysis of citations; (Step 5) presentation of the results—after a vetting of the documents collected, only articles and reviews were retained, and after a thorough analysis of them, a limited number of documents was retained for each year of interest: 34 (2020), 25 (2021) and 28 (2022).

Considering the “explosion” of scientific publications in the last three years related to both the preparation methods and the use of NCs in medical applications, we considered it appropriate to bring to the attention of researchers this comprehensive overview that highlights the significant progress recorded. Starting from the sources and isolation methods of the three types of NCs (cellulose nanocrystals, cellulose nanofibrils and bacterial nanocellulose), their main intrinsic properties were further evaluated with reference to morphological aspects, crystalline structure and their mechanical and biological properties. In addition, we aimed to attract readers’ attention and inform them about the latest developments in the medical fields of major importance. In this regard, an overview of the recent literature on NCs-based hydrogels for TE, DD and WD applications is presented, highlighting the main research directions recorded in these medical fields. The information is presented as a correlation between the methods of obtaining NCs and their effects on the structure and characteristics of NCs-based hydrogels, aspects that ultimately control the efficiency of their use in specific applications. Their main limitations and disadvantages were also pointed out, as well as the future prospects regarding the development of NCs-based hydrogels for medical applications.

## 2. Sources and Isolation Methods for Nanocellulose Materials

NCs include all cellulose-based particles (i.e., at least one dimension in tens of nanometers) having various shapes, sizes, surface chemistries and properties [7]. Considering their sizes and functions, which in turn depend on the source and processing conditions, NCs can be classified into three main subcategories (Table 1): short, rigid cellulose nanocrystals (CNCs); long flexible cellulose nanofibrils (CNFs); and highly crystalline pure bacterial nanocellulose (BNC) [8,9,10,11].

These three types of NCs resemble each other by having a relatively close chemical compositions, but they clearly differ in particle size, degree of crystallinity and morphological properties [26]. These specific characteristics, which distinguish them from one another, crucially depend on the source of origin and the processing technique of the materials. Thus, considering the essential importance of these two parameters, they are presented below, detailed for each type of nanocellulose.

### 2.1. Sources for Nanocellulose Materials

**Figure 2:**
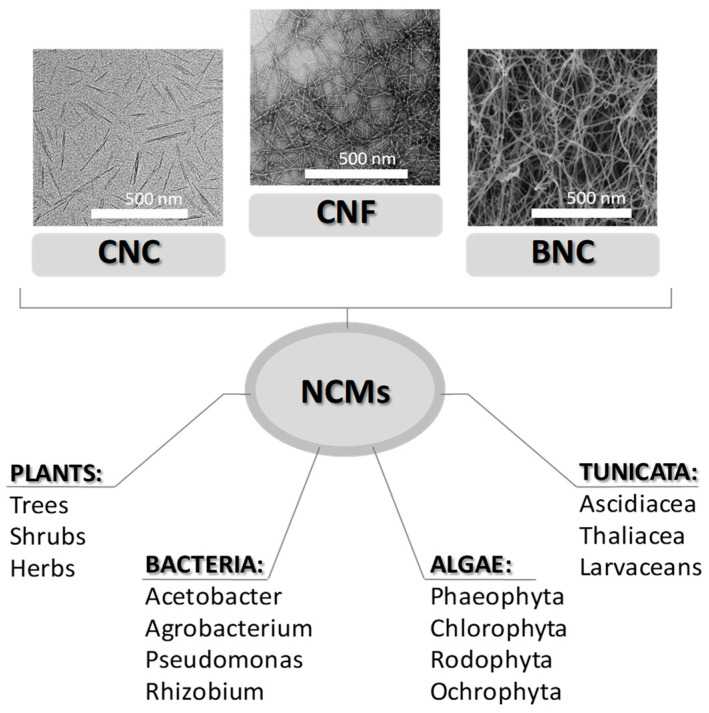
Different sources of NCs and their scanning electron microscopy (SEM) micrographs [30]. Copyright 2021 Elsevier.

One of the main parameters that impact the special characteristics of cellulose nanomaterials, presented in Table 1, is their original source [27,28]. There are four main sources of nanocellulose (Figure 2): plants (trees, shrubs and herbs), bacterial species (*Acetobacter*, *Agrobacterium*, *Alcaligenes*, *Pseudomonas*, *Rhizobium* or *Sarcina*), algae (*Phaeophyta*, *Chlorophyta*, *Rodophyta*, etc.) and animals (Tunicata) [29].

Cellulose nanocrystals (CNCs), also referred to as nanocrystalline cellulose, cellulose nanowhiskers or cellulose crystallites, are extracted from wood or non-woody biomass (agricultural residues and annual plants) by chemically removing (i.e., acid hydrolysis) the lignin, hemicellulose and the amorphous regions of cellulose [31]. Compared to wood, the non-woody biomass contains low amounts of lignin and hemicelluloses, which allows easier access to the cellulose without the use of severe chemical treatments. Another source of CNCs extraction is industrial biowaste, a cheap source with low or even negative costs, which can be a solution to the current environmental problems regarding their elimination from industries [28]. Tunicates are considered the only animal source of nanocellulose, and these are synthesized by some specific enzyme complexes from the plasma membrane of epidermal cells [22]. Tunicate cellulose aggregates are composed of nearly pure cellulose Iβ allomorph [32], and after acid hydrolysis, these yield long nanoparticles with a high aspect ratio, high crystallinity and good mechanical strength [33,34].

Cellulose nanofibrils (CNFs), also known as microfibrillated cellulose, microfibrils or nanofibrillated cellulose, are extracted from wood, sugar beet, potato tuber, hemp, or flax by delamination of the pulp through mechanical treatments (under high pressure), usually after enzymatic prehydrolysis or chemical treatments [10]. CNFs can be biosynthesized in small quantities by brown (*Phaeophyta*), green (*Chlorophyta*), red (*Rhodophyta*), blue–green (*Cynophyta*) or golden algae (*Ochrophyta*) [35,36,37,38], and their structure varies depending on the algae specie. Algae have the advantage of growing faster than terrestrial counterparts, and in addition, they have a low lignin content, which represents an advantage. Besides this, algae are obtained in large quantities as waste from agar production; thus, they can be considered an alternative source for the production of nanocellulose to meet future demands [9].

Bacterial nanocellulose (BNC), also known as microbial cellulose or biocellulose, is a pure component of the biofilm resulting from the activity of aerobic bacteria, such as those belonging to the genus *Gluconacetobacter* [39].

### 2.2. Isolation Methods

Different isolation procedures are used to obtain NCs, depending on their source. These procedures are presented in detail below for each type of nanocellulose discussed: CNCs, CNFs and BNC.

#### 2.2.1. CNCs

**Figure 3:**
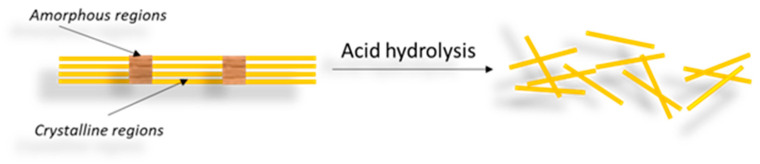
Scheme for obtaining CNCs by acid hydrolysis of cellulose, with the removal of the amorphous region and leaving only the crystalline region.

NCs in nanocrystalline form are one of the most studied types of nanocellulose in term of production methods and their properties [29]. The common method of separating CNCs is controlled hydrolysis of cellulose using mineral acids [12,40,41]. However, this method is part of a multi-step procedure that begins with alkali and bleaching pretreatments and is followed by acid hydrolysis, washing, centrifugation, dialysis, and ultrasonication to form a suspension that may be further subjected to lyophilization [19]. The isolation of CNCs involves turning large pieces of starting material into fine nanoparticles. Thus, at the macroscopic or microscopic level, a transverse cleavage occurs along the amorphous regions of the cellulose, resulting in a rod-like material, as shown in Figure 3 [41,42].

The main sources of CNCs and preparation conditions are presented in Table 2. The isolation of CNCs involves the use of various acids such as sulfuric, hydrochloric, hydrobromic and phosphoric acid, but of these, sulfuric acid is overwhelmingly the most used [29,43,44,45]. The use of different acids produces CNCs with different properties, among which is notably the ability to disperse in aqueous medium, rheological behavior, morphology or crystallinity degree [41]. Besides acid type, the reaction time is also an important parameter with great influence on the crystallinity degree. The longer the contact time, the greater the removal of the amorphous regions within the sample. However, a long contact time can degrade cellulose or even break it down into its precursor sugars. Considering the fact that short reaction times are not enough to obtain nanocrystals, it is obvious that it is necessary to find a balance in order to obtain the final product with the desired properties [40,44].

In the past few years, new routes have emerged regarding CNCs preparation, namely esterification using concentrated organic acids [37], periodate oxidation [13], TEMPO-mediated oxidation [46], reductive amination [47], and microbial or enzymatic hydrolysis [32,48]. The preparation conditions using these types of reactants are also presented in Table 2.

**Table 2:** CNCs sources as well as the preparation techniques and conditions.

Source	Preparation Technique	Preparation Conditions	References
Bleached hardwood pulp	Acid hydrolysis	75% PTA/90 °C/30 h	[12]
Black spruce	Periodate oxidation	NaIO_4/_room T/105 rpm/96 h	[13]
Whatman ashless filter paper	Acid hydrolysis	85% H_3_PO_4_/50 min/100 °C	[45]
	Acid hydrolysis	64 wt% H_2_SO_4_/45 °C/45 min	[14]
	Acid hydrolysis TEMPO-oxidation	2.5 M HCl/70 °C/2 h TEMPO/NaClO	[15]
Tunicates	Enzymatic hydrolysis	Novozym 476 (20 FPU/g); 50 °C/2 h	[32]
	TEMPO-oxidation	TEMPO/NaBr/NaClO	
	Acid hydrolysis	55 wt% H_2_SO_4_/60 °C/20 min	
Red algae	Acid hydrolysis	64 wt% H_2_SO_4_/45 °C/45 min	[37]
Barley straw	Acid hydrolysis	64% H_2_SO_4_/50 °C/75 min	[16]
Ramie fibers	Acid hydrolysis	16 M H_3_PO_4/_150 °C/90 min	[49]
		41–50% H_2_SO_4_/45 °C/30 min	[17]
Bacterial cellulose	Acid hydrolysis	50% H_2_SO_4_/50 °C/40 min	[18]

Abbreviations: PTA—Phosphotungstic acid; NaIO_4_—Sodium (meta) periodate; H_3_PO_4_—Phosphoric acid; H_2_SO_4_—Sulfuric acid; TEMPO—(2,2,6,6-tetramethylpiperidin-1-yl)oxidanyl.

#### 2.2.2. CNFs

**Figure 4:**
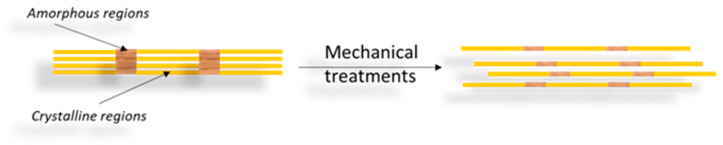
Scheme for obtaining CNFs through a mechanical process of cleaving the cellulose fiber to the nanometric size.

CNFs are the second type of nanocellulose and have a web-like network structure. These are commonly derivatives from natural sources such as wood pulp by mechanical processes (high-pressure homogenization, grinding and refining) preceded or followed by chemical or enzymatic treatments [28,42]. Therefore, the process of obtaining CNFs results in nanofibrils built up by alternating amorphous and crystalline regions (Figure 4) whose cross-sections measure from tens to several hundreds of nanometers, while their length can reach several micrometers [31,40,50].

High-pressure homogenization (HPH) is the most widely used technique for both the laboratory and industrial-scale production of CNFs. However, other strategies are also applied, such as micro-fluidization, micro-grinding, cryo-crushing and ultrasonication [22,31,41]. In addition, different pre-treatments can be utilized before mechanical processes in order to reduce energy consumption as well as to make the surface hydrophobic, such as TEMPO oxidation [51,52], acetylation [53], carboxymethylation [54], alkali pretreatment [55] and enzymatic pretreatment [56]. For instance, an environmentally friendly process of preparing CNFs was reported by Mhlongo and coworkers [57] using industrial hemp (*Cannabis sativa* L.) bast fibers. The process combines the acid hydrolysis treatment with ultrasonication. Besides the fact that low-cost and sustainable industrial waste fibers are used, the obtained CNFs have superior crystallinity and thermal stability.

CNFs were first isolated in 1983 by Turbak and coworkers from bleached softwood fibers using high-pressure homogenization [58]. Additionally, cellulose nanofibers have been obtained from pear [59], Helicteresisora plant [60], oil palm tree [61], banana [62,63,64,65], Citrullus colocynthis seeds [66], cassava peel [67]; hemp [68,69], kenaf [70], wheat straw [71], bagasse [56,70], carrots [72], etc. The main sources of CNFs and their preparation conditions are presented in Table 3.

#### 2.2.3. BNC

BNC is produced by some bacteria in the form of an extracellular material, which is a direct response to their exposure to ultraviolet light, for example, or when defending against fungi, yeasts and other organisms [22]. These bacteria are able to directly produce cellulose microfibrils through microbial fermentation, but without the hierarchical order found in plant cell walls [43].

Some microorganisms with the ability to produce BNC have been reported, namely *Acetobacter (A.) xylinum*, *Salmonella* spp. and *Escherichia coli*. In addition to these, nanocellulose fibers produced by the interaction of acetic acid bacteria and yeast through the kombucha fermentation process, known as Symbiotic Culture of Bacteria and Yeast (SCOBY), can also be mentioned here [74,75,76]. SCOBY fibers have significant potential and are suitable for various applications (i.e., food, pharmaceutical, textiles, cosmetics) due to their special characteristics, such as strong gel film, high elasticity, and optimal deformation and comfort properties. For instance, SCOBY fibers are considered a potential substitute for cotton, a raw material for fabrics, because they have a flexible texture and are brown like synthetic leather. Moreover, they can be considered cheap and environmentally friendly fibers due to their high degradation rate in the environment [75].

**Figure 5:**
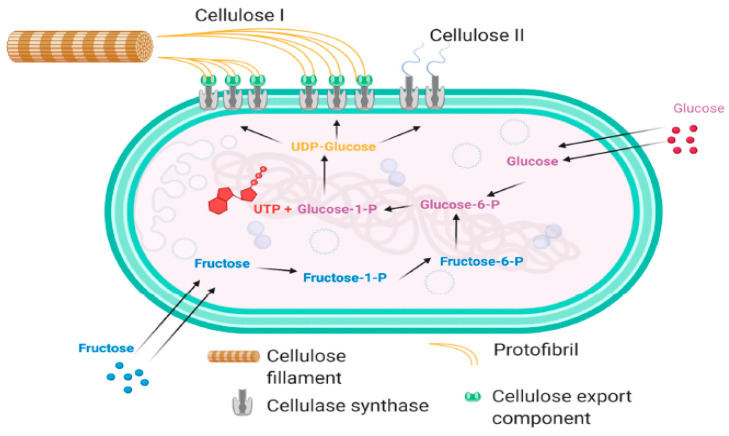
Schematic diagram of the biosynthesis of bacterial cellulose I and II from glucose and fructose [81]. Copyright 2021 MDPI.

However, the bacterium *A. xylinum* continues to be the highest producer of BNC so far [77]. *A. xylinum* is unique in its family for being able to convert carbohydrates into acetic acid during bacterial growth and cellulose production [42,78]. These acetic acid bacteria secrete through their tiny pores located on the cell membrane an abundant 3D network of cellulose fibrils under aerobic conditions, using glucose as a carbon source (Figure 5) [79]. A single *A. xylinum* cell may polymerize up to 200,000 glucose molecules per second, with cellulose synthase or terminal complexes presented in pores on the cell surface and then extruded into the surrounding medium [80]. The cellulose excreted by *A. xylinum* has a chemical structure identical to plant cellulose [23], but it has the advantage of being pure cellulose and therefore does not require rigorous processing (i.e., chemical treatments) to eliminate undesired contaminants or impurities such as pectin, hemicellulose and lignin, as is the case with plant cellulose [41].

Most bacterial cellulose is produced via the conventional static fermentation technique, whereby the bacteria can grow in shallow containers of semi-defined growth medium, in a static incubator at 30 °C, for 7 to 14 days. BNC accumulates at the air–liquid interface as a thick, leather-like, white pellicle that can be easily harvested from the liquid surface interface [81]. Another fermentation technique is the dynamic one (with continuous stirring), in which BNC is obtained dispersed in the culture medium in the form of irregular pellets or suspended fibers [82].

The production parameters, including temperature, pH, culture medium, inoculum ratio and incubation time, should be optimized using readily available and cheap raw materials for the production of high-quality and cost-effective BNC with high yield [83]. Besides the selection of microorganisms and the fermentation method (static or dynamic), the choice of culture conditions has a high impact on BNC production [84]. Studies have shown that up to 30% of the cost of the fermentation bioprocess is due to the fermentation/culture medium, whose composition (i.e., glucose, fructose, etc.) influences the efficiency of bacterial nanocellulose production. Therefore, a high concentration of sugars is necessary for the culture media to improve productivity, which ultimately increases the overall bioprocess cost [85]. Residual products from the dairy industry, wheat straw, fruit juices, rotten fruit, molasses, wine fermentation broth and others have also been used as a source of nutrients for low-cost BNC production [86]. Thus, several researchers have evaluated new sources of carbon and cultivation conditions to optimize BNC production and achieve a more significant yield with reduced costs and production time, and some of the results obtained are presented in Table 4.

## 3. Morphological Aspects and Specific Properties of NCs

Nanocelluloses (NCs) possess unique physical, chemical and biological properties. Generally, NCs are relatively low-cost materials with high availability and renewability. However, at the same time, they have biocompatibility, low or no toxicity, immunogenicity, excellent mechanical strength, outstanding reinforcing potential, and adjustable morphological, electrical, thermal, optical and barrier properties [20,82,91]. As previously presented, the three types of nanocelluloses (CNCs, CNFs and BNC, respectively) are chemically similar but have dissimilar physical characteristics, namely: CNCs—stiffer and highly crystalline rods (90%); CNFs—micrometer-length spaghetti-like fibrils with highly entangled networks and both crystalline and amorphous domains; and BNC—much finer ribbon-shaped fibrils that associate as bundles [92]. Given the fact that there are various sources of cellulose with different physicochemical properties, it is expected that NCs with different characteristics will be obtained, which are reflected in variations in the degrees of crystallinity, polymorphism (cellulose I–IV), the shape of the particles (i.e., aspect ratios) and cross-sectional morphologies [9].

The adequate characterization of NCs is of paramount importance given the fact that these nanomaterials are the basis for the development of well-defined materials with controlled properties that can be used in specific biomedical applications.

To explore their attractive intrinsic properties, NCs have been characterized by a variety of techniques, which allow for the determination of their viscosity, storage modulus, fiber length, width and aspect ratio (length/width), degree of aggregation, crystallinity, and thermal and chemical stability; all this is in order to establish their appropriate application [11,92]. The most common techniques used to investigate and characterize the properties of nanocellulose materials are presented in Table 5.

In the next part, the morphological aspects and the specific properties of NCs that have a great impact on their applicability potential in the medical field are analyzed in detail. Some of the most important properties are of course the biological ones, but neither the mechanical, rheological or thermal properties can be neglected, their identification having an essential role in understanding the behavior of NCs-based materials used in medical applications.

### 3.1. Morphological Aspects of NCs

Considering the undeniable influence of NCs’ morphology on their biological properties, which are decisive in terms of their application in the medical field, we considered it necessary to discuss this, particularly how their morphology is influenced both by the nature of the cellulose sources and by the NCs’ separation or extraction processes. Generally, the morphological structure of NCs varies significantly, taking into account the sources and the extraction processes, which are highly dependent on the efficient removal of non-cellulosic parts (lignin, hemicelluloses, pectin and waxes) and of the amorphous region from the cellulose [93,94].

**Figure 6:**
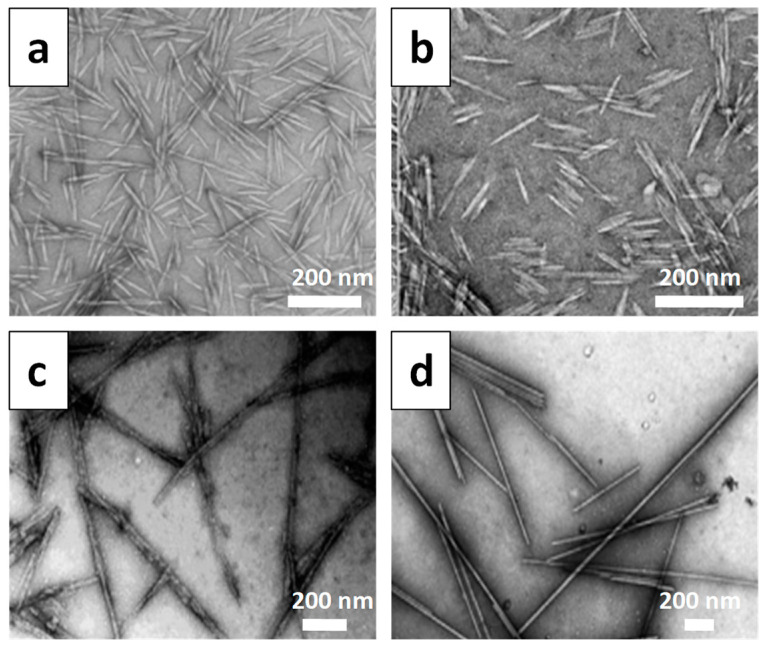
TEM morphology of CNCs isolated from different cellulosic sources: (**a**) wood; (**b**) cotton seed hulls; (**c**) bacteria; (**d**) green algae [95]. Copyright 2021 Wiley-VCH GmbH.

As for CNCs, their geometrical characteristics (i.e., size, dimensions, shape) depend to a certain extent on the cellulose source; however, they depend to the greatest extent on the hydrolysis conditions [92]. Thus, the time and temperature of the hydrolysis process, the acid-to-fiber ratio, and the acid concentration play the most important roles in the morphology and dimensions of the obtained crystals. Increasing the hydrolysis time and the acid/fiber ratio causes a reduction in nanocrystal sizes [28]. An example of the influence of the cellulose source is that of the CNCs obtained from wood, which have widths in the range of 5–40 nm and lengths of 100–300 nm, respectively [12,13]. At the same time, CNCs acquired from non-wood plant fibers (i.e., cotton, wheat, rice or barley straw) were found to have widths of 7–25 nm and lengths of 84–800 nm [14,15,16], while those extracted from ramie fibers have widths of 12–21 nm and lengths of 107–215 nm [17]. Ocean creatures, for example tunicates, deliver nanocrystals with widths of 15–20 nm and several micrometers in length [32]. CNCs from bacterial cellulose, by H_2_SO_4_ hydrolysis, were found to have their width and length in the range of 25–30 nm and 300–400 nm, respectively [18]. To precisely illustrate these dimensional differences, Figure 6 presents some TEM images representing CNCs obtained from four different cellulosic sources, namely wood, cotton seeds, bacterial and green algae, respectively.

**Figure 7:**
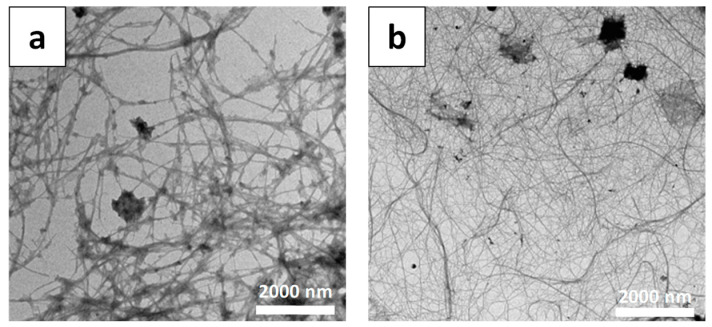
TEM images of CNFs obtained from banana peels by (**a**) chemical and (**b**) enzymatical treatment [97]. Copyright 2014 Elsevier.

In contrast to CNCs, CNFs show rather high contents of amorphous regions, and the chains are significantly longer, up to several μm, while the widths vary from ten to several hundred nanometers (20–100 nm) [11,19]. Usually, CNFs sizes vary depending on the pretreatment (chemical or enzymatic) and mechanical treatment applied to the cellulosic substrate (Figure 7), but the determination of CNFs length has remained a challenge due to the entanglement of the nanofiber and the difficulties in identifying both ends of individual nanofibers [96]. CNFs display a high aspect ratio and can easily form web-like structures, contrary to CNCs particles [92]. For instance, as seen in Figure 7, the pretreatment of banana peel fibers, chemical (KOH/NaClO_2_/pH 5.0/70 °C) or enzymatical (xylanase 50 U/g) affect the fibers’ dimensions. Thus, the milder method with enzymes provided longer nanofibers with higher aspect ratios, while, on the other hand, the chemically treated banana fibers had 300% larger crystallinity, as compared with the 200% higher crystallinity of the enzymatically treated samples. Therefore, the chemical method removed amorphous components more effectively than the enzymatical one and enhanced the crystalline content in the sample [97].

**Figure 8:**
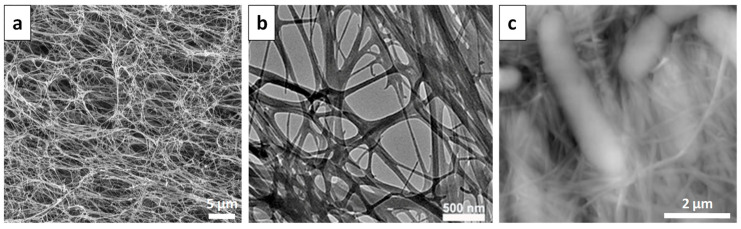
Micromorphology of BNC: (**a**) SEM image of freeze-dried BNC [98], Copyright 2006 Elsevier; (**b**) TEM image of natural BNC [99], Copyright 2022 MDPI; (**c**) AFM image of BNC [100], Copyright 2005 Wiley-VCH GmbH.

Structurally, BNC is similar to CNFs, but its elementary nanofibrils have a lateral size of 8–10 nm and are aggregated into nanofibrillar bundles (microfibrils) (Figure 8) having a width of 50–150 nm, which in turn are interlaced with one another and form a three-dimensional network [11].

Mainly, the supramolecular structure of BNC and its physical properties are directly influenced by its production technique [23]. For instance, the static fermentation of BNC, which is the standard cultivation method, results in a highly homogeneous supramolecular structure characterized by uniaxially oriented ribbons. Instead, dynamic fermentation leads to the obtaining of BNC in the form of large spheres, with a disordered, curved, and overlapping ribbon-like morphology [23]. Differences were also observed regarding the mechanism of cellulose biosynthesis or the organization of cellulose synthesis’ sites by different bacteria, i.e., the width of cellulose fibrils in *Asaiabogorensis* and *Gluconacetobacterxylinus* ranges from 5 to 20 nm and from 40 to 100 nm, respectively [80].

In conclusion, it can be stated that depending on the desired characteristics of the NCs and the chosen field of application, the morphology of the NCs can be tailored, either by using a certain source of cellulose with specific characteristics or by choosing a special separation technique.

### 3.2. Crystalline Structure of NCs

Besides morphological characteristics, the crystallinity degree of NCs is another parameter that has a major influence on their properties, its control having a strong impact on their subsequent application in the medical field. More precisely, their crystallinity affects the mechanical and biological properties, the dissolution process, the total amount of absorbed water, and so on. For instance, NCs with a higher crystallinity degree will present exceptional mechanical properties, a fact that will be discussed in more detail in the following subsection. The high crystallinity and the hydrogen bond network present in the nanocellulose’ structure make NCs insoluble in water and in most of the organic solvents [101]. In addition, the swelling degree of NCs-based hydrogels is affected by the crystallinity degree of NCs due to the fact that the amorphous region is more hydrophilic than the crystalline region [102]. Moreover, the in vitro pro-inflammatory and pro-oxidative effects of NCs have been correlated with their higher crystallinity degrees [103].

CNCs have high crystallinity (90% or greater) since most of the amorphous portion of the cellulose is removed during hydrolysis [30]. A number of parameters, such as the source of cellulose, isolation process conditions and various pretreatments, determine the final crystallinity of nanocellulose in crystal form. For instance, if hydrolysis conditions are discussed, the crystallinity of CNCs is influenced by the type of used acid; H_2_SO_4_ and HCl hydrolysis determine a crystallinity of 72 and 84%, respectively [104]. By varying the hydrolysis time from 20 to 40 min, the crystallinity of kenaf bast CNCs was boosted from 75 to 81%, whereas further increasing the time to 120 min reduced the crystallinity to around 75% because of the destruction of the crystalline part [105].

CNFs obtained under milder conditions of mechanical treatment of cellulosic biomasses present both crystalline and amorphous regions, and as a result, they present a lower crystallinity degree with respect to CNC or BNC [7]. The crystallinity and crystallites’ size of CNFs depend on the cellulose source, on the number of passes through the high-pressure homogenizer [106], and the use of pretreatments such as enzymatic hydrolysis [56], TEMPO oxidation [52] or carboxymethylation [54].

The nanocelluloses from bacteria (BNC) have a crystallinity higher than 80%, which results in a high thermal stability and a degree of polymerization higher than 20,000 [7]. Generally, BNC is composed of cellulose type Iα and Iβ, with a proportion that varies due to different culture conditions. Thus, this ratio can be modified if one changes the nutrient medium, type of reactor, pH or bacterial strain [80,107].

It can be concluded that the crystallinity of NCs is a variable structural factor and is responsible for the performance of the material that is being designed and must be taken into account as an important characteristic when we talk about the application of NCs in the medical field.

### 3.3. Mechanical Properties of NCs

NCs contain regions of ordered crystalline and disordered amorphous regions in different proportions that affect their mechanical characteristics [42]. The amorphous regions determine the material flexibility and plasticity, while the ordered regions contribute to the rigidity of the material [108]. Considering that different types of NCs show variations in their crystallinity, it is expected that they will have different mechanical properties. Literature data have demonstrated that NCs present high strength and stiffness, which makes them suitable to be used as a promising reinforcing element in the preparation of different three-dimensional networks.

CNCs’ mechanical properties are the result of their high crystalline order and well-defined size and morphology [7]. Thus, CNCs present exceptional mechanical properties because of the removal of the amorphous parts of the nanofibers, which provides a strengthening effect. Its values for tensile strength (7500–7700 MPa) together with Young’s modulus (130–250 GPa) are comparable to the carbon fiber of TORAYCA^®^ T1000G, one of the world’s best materials best for its mechanical properties [78].

CNFs present crystalline and amorphous regions and have the morphology of a network with lots of entanglement points. Consequently, CNFs material has more flexibility than that of CNCs, which allows for more elongation during any mechanical strain [40]. The mechanical properties of CNFs are strongly associated with the chemical composition of their origin resources. For example, CNFs from kenaf, hemp and cotton contain more crystalline cellulose, leading to excellent tensile strength and modulus and higher thermal stability, while CNFs extracted from bamboo, oil palm and wheat straw with higher amounts of hemicelluloses and extractives are more amorphous and have weaker mechanical strength and degrade at a lower temperature [28].

The structural singularity of the BNC fibrillated network results in unique mechanical characteristics, which are found to be superior to those of plant-origin cellulose [6,78,109]. Never-dried BNC has outstanding mechanical properties with a stress–strain behavior that seems like soft tissue. The tensile strength of BNC pellicle has been reported to be 2 MPa, which yields incredible performance considering its 99% water content [110]. However, in the dry state, due to its crystalline nanofiber structure, BNC demonstrate remarkable mechanical properties, i.e., a single fiber of BNC is equivalent to steel and Kevlar [100]. The elastic modulus of BNC has been reported to be 78 GPa, the value being slightly higher than that of glass fiber (70 GPa) [24].

Taking into account all these different mechanical characteristics of NCs and their subsequent medical applications, it can be easily predicted whether a certain type of nanocellulose will lend itself to that application or can be intervened in a certain way to modify it (i.e., functionalization, grafting, mechanical reinforcement, etc.), making it the perfect material for the desired field.

### 3.4. Biological Properties of NCs

One of the main requirements of any material for medical applications is its biocompatibility, which is the ability to remain in contact with living tissue without causing toxic effects on the biological system [6]. Toxicological testing of NCs has proved to be particularly difficult considering the wide variety of resources, the different methods of preparation and the different physicochemical properties of the three types of NCs, which must be taken into account when evaluating the toxicological properties.

Overall, NCs are often considered materials with no cytotoxicity or with low cytotoxicity and immunogenicity, but this is not entirely true. Indeed, there is no evidence of a serious influence of these materials at the cellular level or in in vivo animal experiments. However, numerous studies have shown that this is only valid at low concentration ranges (50–100 mg/mL), whereas at high concentrations (>100 mg/mL), cytotoxic effects or even cell death or changes in the gene expression of cells are recorded [111,112,113,114,115,116,117,118]. Many studies have confirmed the significant influence of NCs concentration on cytotoxic character, regardless of their type, morphology, source of origin or type of cells used. However, an imperceptible separation can be made between BNC and the other two nanocelluloses, CNCs and CNFs, most likely due to the different method of separation (i.e., the use of chemicals or not), being the main starting point of their slightly different properties.

For instance, CNCs originating from different sources (i.e., softwood pulp [117,119], cotton cellulose [120], raw rubberwood or Kenaf-bast fibers [115]) and used in a wide range of concentrations (15–300 µg/mL) showed negligible or no cytotoxicity at lower concentrations (<50 µg/mL) without triggering any inflammatory response or causing damage to the cell membrane. However, at concentrations higher than 100 μg/mL, CNCs induced a slight decrease in cell viability, and in some cases, a 55% cytotoxicity has even been reported [120]. On the other hand, in vitro cytotoxicity tests with CNFs showed no evidence of toxic behavior on the cell membrane and no oxidative stress, but at a higher concentration (>250 µg/mL), a slight inhibition of the metabolic activities of the cells and consequently an inhibition of their proliferation was recorded [121,122,123,124]. As is observed, the concentration at which a slight cellular inhibition occurs is higher for CNFs than CNCs. Thus, a more suitable biocompatibility was recorded for CNFs compared to CNCs, which may be due to the mild chemical treatments applied in CNFs separation techniques, which provide nanofibers with a non-toxic surface against which the cells demonstrate higher affinity [125]. Regarding BNC, in vitro cytotoxicity studies showed no cytotoxicity, and BNC did not induce any DNA damage, apoptosis, or necrosis in cells, regardless of the concentration used (100, 500 or 1000 µg/mL) [126].

In addition to concentration, several other parameters can be mentioned as being decisive in the establishment of the in vitro cytotoxic effects and inflammatory responses upon exposure to different NCs, such as the particle size and shape, the surface charge and chemistry, and the morphology of each NC. However, cell type cannot be neglected either. Each cell type has a different function and consequently has a different response when exposed to NCs. Thus, a type of cell exposed to NCs will have a certain response, different from another type of cell with a different function but exposed to the same type of NC [127].

Regarding the charge-dependent cytotoxic effect of NCs, the results are also different in this case, depending on the nanomaterial type. For example, in the case of CNCs derivatized with different carboxyl contents varying from 6.6 to 1.7 mmol/g, a charge-dependent decrease in mitochondrial activity for CNCs with a high charge density was recorded [117]. On the other hand, the introduction of charged groups on the CNF’s surface (i.e., carboxymethyl or hydroxyl-propyl-trimethyl-ammonium) did not induce cytotoxicity, the metabolic activity of cells not being significantly affected at a concentration range of 50–500 μg/mL, and no signs of toxicity were observed in terms of cell membrane damage. Thus, regardless of the type of charged groups for surface functionalization, CNFs showed low cytotoxicity, with a maximum of 10% [128].

Similar to the in vitro toxicity of NCs, studies regarding in vivo toxicity have shown that it depends on several parameters that must be taken into account, such as the nanocellulose type (e.g., different morphology, crystallinity, presence of functional groups), the exposure method (i.e., ingestion, direct skin contact or inhalation), the exposure duration (24 h, a few days or weeks) or the dose of NCs administered (single or repeated dose). For instance, the influence of NC type on its cytotoxicity and immunogenicity was confirmed by in vivo experiments performed on mice when observing different immune responses to CNFs and CNCs; namely, a significantly increased inflammatory response of proinflammatory cytokines and chemokines was observed from their exposure to CNCs compared to CNFs [129]. Regarding the influence of morphology and particle size for the same type of nanocellulose, Yanamala and coworkers [130] showed that the particle dimensions of the CNCs may be a critical factor affecting the cell inflammatory responses. Thus, by using two processed forms of CNCs, i.e., a gel/suspension and a freeze-dried powder, respectively, different toxicities expressed by an increase in leukocytes and eosinophils in the lungs was registered as a result of differences in the CNCs particle dimensions; biomarkers for tissue damage were elevated to a higher extent in the mice exposed to CNCs in powder form, while CNCs gel/suspension form caused a significant increase in the accumulation of oxidatively modified proteins.

A remarkable difference in the in vivo NCs toxicity was observed when they were administered in rats by different techniques. After introduction into the lungs by oropharyngeal aspiration, CNFs turned out to be the nanocellulose that caused an accentuated immune response compared to CNCs [131]. The pulmonary exposure to CNFs induced pulmonary inflammation and genotoxicity and a systemic acute phase response. However, the same thing does not happen when a 1% (*w*/*w*) CNFs suspension is administered to Wistar rats by the gavage technique two times a week for five weeks. In this case, no significant differences in hematology, serum markers, or histology were observed between exposed rats and controls. These results suggest that CNFs administered orally (ingested) has little acute toxicity and is probably harmless in small quantities [132]. Moreover, the CNFs-induced systemic acute phase response could be reduced through functionalization by the carboxylation of OH groups. Thus, carboxylated CNFs produced lower neutrophil influx and systemic SAA3 levels, suggesting that the carboxylation of the OH groups may be considered a strategy to lower the toxicity of these materials [133]. However, it has been noted that CNFs materials do not induce long-term pulmonary inflammation, but still, non-functionalized CNFs are more prone to trigger inflammatory reactions in vivo than CNFs functionalized with anionic groups [134].

## 4. Biomedical Applications of Nanocellulose Hydrogels

### 4.1. NCs-Based Hydrogels—Key Aspects

Hydrogels can be defined as three-dimensional networks formed by the crosslinking of hydrophilic polymers through physical and/or chemical bonds that have the ability to absorb a large number of water molecules without losing their integrity [135]. Natural polymers can be easily engineered to produce hydrogels that outperform synthetic polymers in terms of biological performance, extracellular matrix similarity, and diverse and highly selective coupling chemicals. In this regard, NCs-based hydrogels are attracting much attention due to their diverse properties, such as biodegradability, renewability, biocompatibility, flexibility, their high degree of crystallinity, and their unique surface chemistry [108]. Besides that, nanocellulose and its derivatives can efficiently impart mechanical reinforcement, exhibit abundant hydrophilic functional groups such as hydroxyl, carboxyl and aldehyde groups, and tends to generate hydrogels with exceptionally high-water contents (>90 wt%) due to their high surface-to-volume ratio [136].

**Figure 9:**
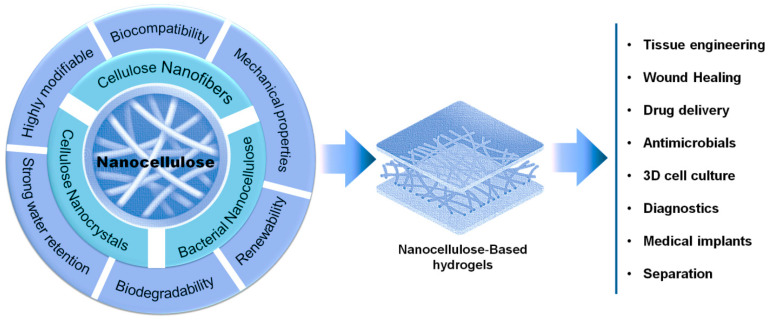
Properties and biomedical applications of nanocellulose.

As a consequence, these types of materials have yielded excellent progress in research and development, particularly in the areas of tissue engineering, regenerative medicine and drug delivery (Figure 9). This initiative also aligns with the growing demand for sustainable materials to advance healthcare and biomedical technologies aimed at improving well-being and extending human lifespan [137].

In the development of hydrogels, all three types of nanocellulose—CNCs, CNFs, and BNC—can be employed. In this regard, NCs and their derivatives can be used as a single component to form hydrogels, aerogels or nanogels or can be dispersed in 3D structures to form composite matrices and hybrid gels. Due to the fact that cellulose-based hydrogels are insoluble in water, CNFs, BNC, and CNCs form a colloidal suspension in water as opposed to other polymer systems, which form solutions. Hence, the 3D network is composed of fibril entanglement and physical interactions such as hydrogen bonds and electrostatic, van der Waals, hydrophobic and ionic interactions. Chemical crosslinking such as radical polymerization, addition and condensation reactions, high-energy radiation and enzymatic reactions can further strengthen the network by creating covalent bonds. In addition, NCs can be used as such or can be chemically modified with functional groups or by grafting biomolecules such as enzymes, antibodies and peptides. This functionalization provides for the physical and chemical improvements of its properties as well as control over biological interactions, thus obtaining hydrogels for specific applications [138].

CNCs represent one of the best choices in terms of surface modification due to properties such as high purity and crystallinity. Most studies, however, concentrate on their potential to act as fillers or reinforcing agents because of their low ability to entangle [139]. CNFs can impart high mechanical strength and tensile elasticity to hydrogels due to their high degree of polymerization, excellent water absorption capacity, and flexibility. CNFs have also been shown to increase the viscosity of hydrogels and improve their printability [140]. At the same time, BNC finds applicability in the preparation of hydrogels due to their biocompatibility and high mechanical strength, but also due to their fibrous morphology, which allows for gas and nutrient exchange, and unique network resembling the natural extracellular matrix (ECM) [141]. Subsequently, all reported studies on their toxic effects on cells and organs classified hydrogels based on CNFs, CNCs, and BNC as non-carcinogenic and non-cytotoxic [108,124].

There are numerous original papers and academic reviews on the properties and applications of NCs-based hydrogels [91,137,141]. Only recent studies on their potential applications in wound healing, tissue engineering, and drug delivery will be considered in this section of the review (particularly for the reports of the past 3 years).

### 4.2. Wound Dressings

Different traumas or skin conditions or even microbial contamination, which are obtained after affecting a certain area of the skin, are considered wounds [142].

The material considered “ideal” for treating wounds should absorb exudate and toxins, be sterile and capable of maintaining a suitable humidity between the injury and the dressing, have good permeability to gases, protect the wound site from the external environment, accelerate the healing process, and be easily removed without causing additional harm to the skin [143].

NCs-based hydrogels could be an ideal choice as a material for wound treatment because they fulfil the necessary requirements and, moreover, demonstrate other unique characteristics, such as softness, malleability and a fibrous morphology (i.e., CNFs and BNC) similar to the extracellular matrix providing an ideal environment for cell growth and skin regeneration [141,144]. Likewise, CNCs are typically used as additives in hydrogels owing to their discrete nanoparticulate morphology.

An advanced wound management strategy is the encapsulation of antimicrobial agents in hydrogels for bacteria-associated wounds as studies show, for example, that subsequent infections account for more a than 75% morbidity rate in burn patients [145]. For instance, antimicrobials such as antibiotics (levofloxacin [146], bacitracin, amoxicillin [147], vancomycin, ciprofloxacin [148], ofloxacin [149], clindamycin hydrochloride [150]), silver nanoparticles [151,152], metal cations [153], and natural compound (thymol [154], alpha-tocopherol [155], propolis [156], curcumin [157], oregano essential oil [158], vitamin C,E [159], diosgenin [160] and resveratrol [161])-loaded NCs-based hydrogels have been reported (Table 6).

In order to increase the contribution of NCs in wound healing, or more precisely in skin regeneration, different active substances can be added, including growth factors or even certain types of cells [166,167,168].

In a study by Mariia et al., a new hydrogel based on chitosan-ulvan reinforced with cellulose nanocrystals incorporating an epidermal growth factor (EGF) drug (CS-U-CNCs-EGF) was developed through a lyophilization process [169]. The results highlighted the fact that the incorporation of CNCs led to the modification of the porous microstructure and an improved mechanical stress curve, thermal behavior, and swelling. The in vitro study showed that the inclusion of EGF in the hydrogels induced excellent proliferation of L929 fibroblast cells and further promoted significant skin wound recovery during 15 days of in vivo testing. A further novelty was the development of a sprayable hydrogel formulation based on CNCs and tobramycin (TOB), an antibiotic used in the treatment of bacterial infections [170]. The hydrogels were formed by simply mixing the antibiotic and CNCs, which resulted in ionic interactions between the sulfate groups of the polysaccharide and the amine groups of the TOB. The hydrogels showed excellent biocompatibility, tunable mechanical strength, shear-thinning, and fast self-healing properties in addition to prolonged antibiotic release, which further inhibits wound infection.

Intelligent dressings can be defined as multifunctional materials with sensor motifs that respond to specific changes in the wound environment (i.e., pH or elevated protease levels in chronic wounds) and can treat specific pathological issues at the molecular level [171,172,173,174].

**Figure 10:**
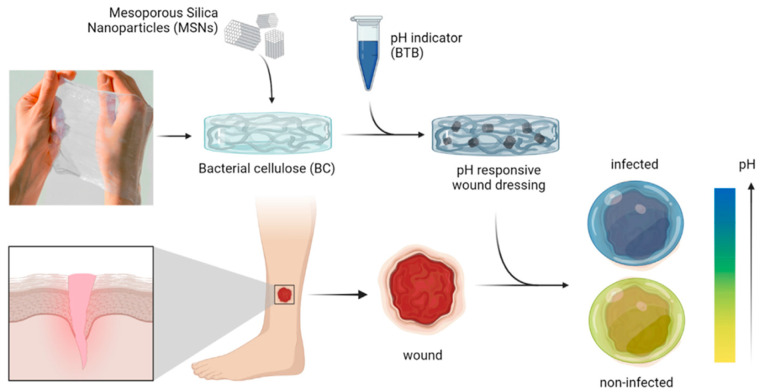
The pH-responsive nanocomposite wound dressings were obtained by impregnating bacterial nanocellulose with mesoporous silica nanoparticles loaded with a pH-responsive dye [175]. Copyright 2023 Elsevier.

Eskilson et al. proposed a new strategy for incorporating pH-sensing capabilities into a hydrogel obtained by self-assembling mesoporous silica nanoparticles (MSNs) into BNC (Figure 10) [175].

The pH of healthy, uninfected skin is known to be slightly acidic, ranging between 4 and 6. At the same time, the pH of chronic wounds is usually in the range of 7–9, and infections have been shown to cause an increase in wound pH. Wound dressings were thus developed for continuous pH sensing by incorporating a pH-sensitive dye into MSNs, respectively bromothymol blue (BTB). BTB recorded a color change with pH variations; namely, a transition from yellow to blue was observed for a pH varying from 5.5 to 8. The hydrogel thus obtained shows a good ability to retain exudate and a cell growth of human primary cultures (keratinocytes and fibroblasts), properties that make it effective for dressing wounds. Furthermore, BNC-MSNs composite hydrogel allowed the assessment of wound status both in vitro and in vivo in an infected porcine wound model.

**Figure 11:**
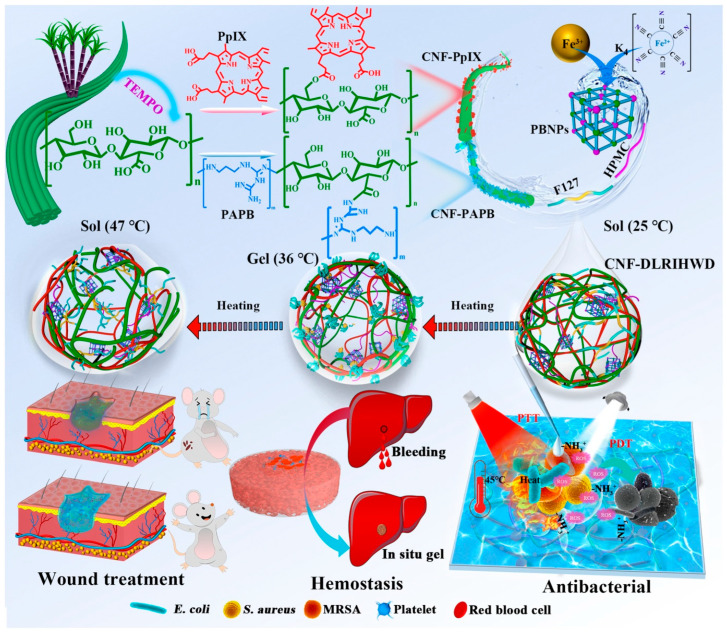
Preparation steps of CNFs-DLRIHWD hydrogel and its application in wound healing [176]. Copyright 2022 Elsevier.

Furthermore, some endeavors have been devoted to the use of CNFs for the development of white light and NIR dual light-responsive hydrogels with rapid hemostasis and antibacterial activity [176]. Photodynamic therapy (PDT) uses photodynamic agents to induce reactive oxygen species, which can kill drug-resistant bacteria, whereas photothermal therapy (PTT), which is induced by near-infrared (NIR) irradiation and converts laser energy into heat energy via photothermal agents, demonstrated deep tissue penetration and excellent exogenous antibacterial and biofilm elimination effectiveness (Figure 11). This was achieved by using white light responsive CNFs-Protoporphyrin IX (CNFs-PpIX) and hydroxypropyl methyl cellulose (HPMC) as the NIR responsive switch, temperature-sensitive switch, and binder, along with endogenous antibacterial CNFs-polyaminopropyl biguanide (CNFs-PAPB) as the skeleton, Prussian blue nanoparticles (PBNPs) and Pluronic^®^ F127 (F127). The resulting hydrogel coded as CNFs-DLRIHWD has an antibacterial activity higher than 99.9% against *Escherichia coli* and *Staphylococcus aureus*. Furthermore, it can cause rapid in vitro blood clotting as well as in vivo hemostasis by producing an in-situ gel and functioning as a physical barrier.

Another interesting hydrogel with dual near infrared (NIR-) and pH-responsiveness for tumors and infected wounds was developed by Chen and coworkers [177]. In brief, CNFs-based hydrogels with nanocage structures were loaded with doxorubicin and indocyanine green, and the studies showed that the dressing has very good photothermal conversion and can effectively eliminate bacterial biofilms and kill A375 tumor cells. This hydrogel can be regarded as playing a pivotal role in the effective treatment of complex wounds.

### 4.3. Tissue Engineering

By using concepts and techniques from engineering, chemistry, biology, and medicine, tissue engineering (TE) has become an established approach to creating biological substitutes for treating, replacing, or regenerating damaged tissue or organs [21]. Furthermore, due of its outstanding properties, nanocellulose has consistently proven to be a promising component in a variety of formulations for TE applications [178,179]. An overview of the latest findings in the field of NCs-based hydrogels that are used for TE applications is summarized in Table 7.

Various fabrication techniques have been developed for TE scaffolds, with three-dimensional (3D) printing receiving particular attention. Additive manufacturing, also known as 3D printing, is a method of generating 3D materials in a digitally controlled layer-by-layer manner that has gained popularity in the last decade for TE and regenerative medicine. It has several advantages over traditional methods, including controlled chemistry, complex internal geometries and the ability to obtain customizable geometries for personalized therapy [198]. In the case of biomedical bio-applications, specialized bioprinters frequently use inks based on hydrogels. Furthermore, studies have confirmed the shear thinning behavior of NCs-based inks as well as their excellent viscoelastic properties, both of which are required for successful 3D printing [199,200]. Numerous studies detail the successful 3D printing of NCs in combination with various polymers, such as gelatin [201], xanthan gum [202], poly(N-isopropylacrylamide) [199], chitosan [203], polyvinyl alcohol [204], pectin [200] tragacanth gum [205], as well as other compounds such as peptides [206], zeoliticimidazolate frameworks [207], hydroxyapatite [208], carbon nanotubes [209], aloe vera [210], quince seed mucilage [211] and so on.

In both in vivo and in vitro investigations, crosslinked sodium alginate and CNFs hydrogel bioinks have demonstrated outstanding characteristics in cartilage tissue engineering for articular, auricular and nasal reconstruction [212,213,214,215]. Furthermore, aerogels with a multiscale pore structure and tunable Poisson’s ratio based on CNFs and polyethylene glycol diacrylate (PEGDA) were successfully obtained for cartilage repair using a combination of stereolithography and freeze-drying. This strategy enabled hierarchical control of the pore structure, and studies revealed that the materials can support stem cell proliferation and differentiation [216].

**Figure 12:**
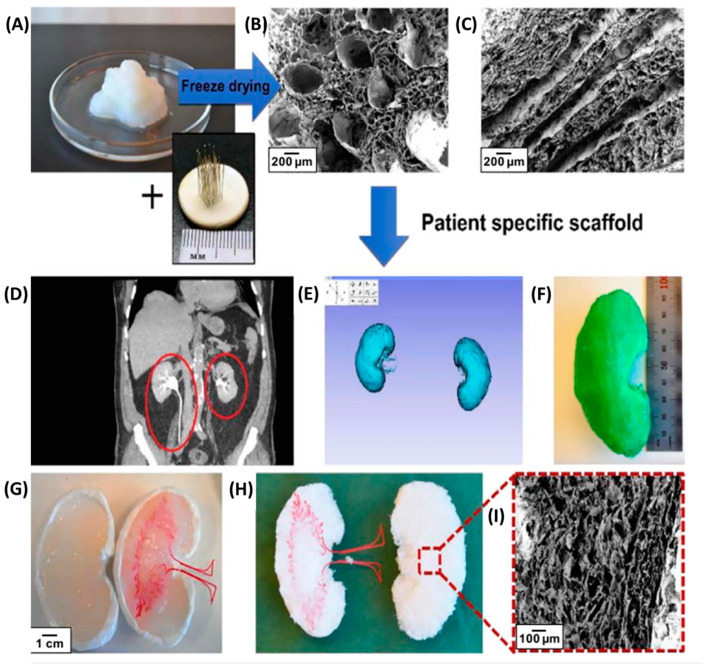
(**A**) BNC blended with alginate, (**B**) SEM image of homogenized BNC freeze-casted around clay-needle construct perpendicular cut to the channels, (**C**) cross-sectional cut of the channels, (**D**) abdominal computed tomography (CT) image with kidneys marked in red. The data have been anonymized, (**E**) STL-file of kidneys displayed in software Slicer, (**F**) printed PLA-kidney template, (**G**) clay-negative filled with homogenized BNC/alginate with an inserted PLA sacrificial template vascular tree, (**H**) freeze-dried BNC/alginate sponge cut in half holding a PLA sacrificial template vascular tree prior to the removal of the template by hydrolysis, (**I**) SEM image of sponge microstructure [217]. Copyright 2019 IOP Publishing Ltd.

Scaffolds based on BNC and alginate were prepared to facilitate reendothelialization by creating a broad structure and a specific nanomorphology of the vascular mimetic lumen [217]. Initially, to demonstrate the vascularization, channeled structures were made inside the BNC hydrogel where human umbilical vein endothelial cells (HU-VEC) were cultured, and further, it was shown that the cells attach in the channels. Next, by using a 3D printer, more complex constructs with interconnected macroporosity and a vascular-like lumen structure have been created, as seen in Figure 12. To cast a kidney mimetic construct of adequate size, the patient’s data obtained from the CT scans were used. Thus, a porous kidney-shaped scaffold was used by adding BNC/alginate gel to the kidney-like construct, which was then freeze-dried. As a result of this study, it was confirmed that 3D printing can be used to make various and complex tissue engineering constructs.

Another area of potential growth is the use of 3D printing to create scaffolds for lung tissue engineering. In this regard, a new ink consisting of regenerated silk fibroin and 2,2,6,6-tetramethylpiperidine-1-oxyl (TEMPO)-oxidized BNC has been developed for extrusion-based 3D printing. The hydrogel exhibited excellent shear thinning behavior with reversible stress softening and was able to mimic the structure of the extracellular matrix of the lung, which further induced lung epithelial stem cell orientation and proliferation even after 7 days of culture [218].

Another field of application in which 3D-printed nanocellulose hydrogels are used is bone tissue engineering. Thus, Imand and coworkers [219] have developed an osteogenic bioink composed of alginate, CNFs and polydopamine nanoparticles. This demonstrated a special printing potential, but also an important osteogenesis, evaluated by in vitro studies of scaffolds loaded with 3D-printed osteoblasts. Another interesting bioink developed for bone tissue engineering was based on an alginate and gelatine that incorporated CNFs and bioactive glass, with studies showing that the polysaccharide enhanced the rheological properties, resulting in high printability without cell damage and improved osteogenic activity [220].

### 4.4. Drug Delivery

**Figure 13:**
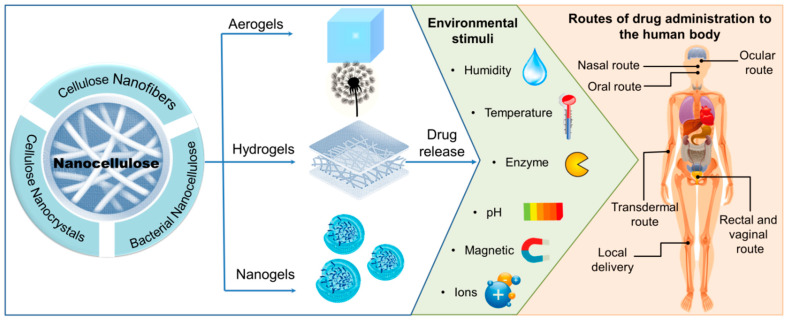
Main recent research directions for NCs-based hydrogels in drug delivery.

Various drug delivery (DD) systems based on NCs hydrogels, aerogels or nanogels have been developed in recent years [29]. Thus, Figure 13 summarizes the main recent research directions for NCs-based hydrogels in drug delivery. CNFs, BNC, and CNCs all benefit from biocompatibility, tunable surface chemistry, open pore structure, and a high surface-area-to-volume ratio, which may allow for higher levels of drug binding and thus higher bioavailability [221]. However, their hydrophilic character makes them able to bind only with water-soluble drugs. As a result, a variety of surface modifications or pretreatments have been performed to improve binding to hydrophobic drugs, such as covalent chemical modification or the use of surfactants [21]. A review that is worth mentioning and that deals extensively with this topic was recently carried out by Tortorella and coworkers [222].

Nanocellulose has proven to be an effective drug carrier for oral, ocular, nasal, transdermal, intratumoral, and local administration, and Table 8 summarizes the most recent research in this area. In addition to conventional drugs, NCs-based hydrogels are also capable of delivering nucleic acids, proteins, plasmid or genes [223,224].

Nevertheless, controlled and targeted delivery is considered the most effective approach for the administration of bioactive substances. The biopolymer can influence how drugs are released into the body through a variety of strategies that include water retention, film formation, and rheology control [242]. However, more complex release systems that can regulate the release of pharmaceutics in response to specific conditions or stimuli, such as temperature, pH, enzymes, magnetic or electric fields, ions, or glucose, have been the subject of recent research. When the external environment changes, the responsive hydrogel will degrade, shrink or expand as needed, allowing the release of loaded drug molecules [243,244].

Recently, Liu and coworkers [238] developed mesoporous polydopamine nanoparticles wrapped with graphene oxide, which were physically cross-linked in CNFs hydrogel. The nanoparticles exhibited a high drug loading ratio (up to 35 wt% for tetracycline hydrochloride), and its release rate could be accelerated by near-infrared (NIR) light irradiation or by changing the pH value. Emam and Shaheen [227] studied a dual-responsive hydrogel in terms of simultaneous pH and temperature responsiveness by using succinylated cellulose nanocrystals and poly(N-isopropylacrylamide) (PNIPAm) obtained by radical polymerization. These hydrogels respond to pH and temperature in terms of their swelling capacity. Thus, the studied material swells at low temperatures and shrinks as the temperature rises. Furthermore, decreasing the pH from 10 to 2 increases the release percentage of the drug (Famotidine).

**Figure 14:**
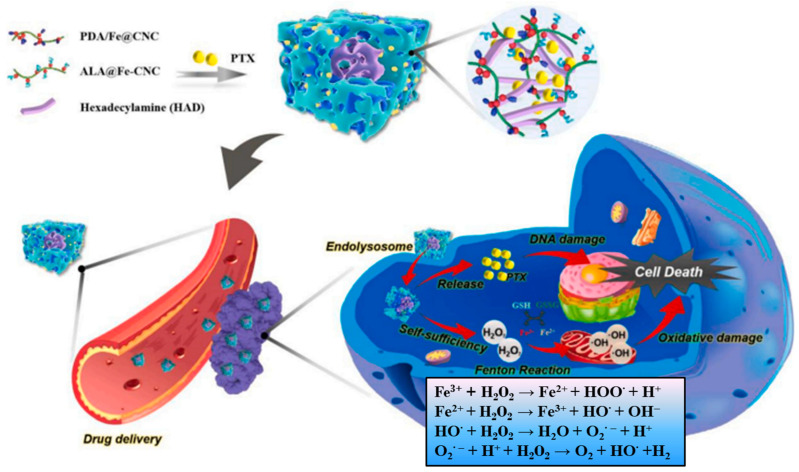
Design of the PTX-PDA/ALA/Fe@CNCs nanohydrogel, and Fe^3+^-activated Fenton reaction to increasing ROS concentration in tumor cells [236]. Copyright 2021 Elsevier.

Another pH-sensitive nanohydrogel based on CNCs modified with 5-aminolevulinic acid (ALA/Fe@CNCs) or dopamine (PDA/Fe@CNCs) through the coordination of iron ions was developed by You and coworkers [236] for the co-administration of a Fenton-like reaction system and an antitumor drug (paclitaxel, PTX). The design of the metal-cellulose network nanostructure, which allowed the combination of ROS-mediated oxidative damage with chemotherapy, is shown in Figure 14. The results revealed that the new nanogels are biocompatible and have excellent antitumor performance.

Several additional studies have confirmed the suitability of NCs hydrogels as stimuli-sensitive systems for the controlled release of drugs, paving the way for new accessible formulations aimed toward complex therapies [228,229,234,237,245].

## 5. Limitations and Future Aspects

NCs-based hydrogels have emerged as promising biomaterials for a large number of applications, but nevertheless, these materials also have limitations and challenges that must be addressed to realize their full potential in the medical field. In this sense, this part of the review aims to discuss their limitations and potential future directions for their development.

It is clear that more thorough research is still required to completely comprehend the processes by which NCs interact with biomolecules and to comprehend the long-term implications of these interactions in the human body. Namely, detailed in vivo and long-term biocompatibility studies are needed. Due to differences in the various characteristics of all types of NCs, such as preparation methods, source materials, surface modifications as well as the type of animal model employed (for example, species, dose and duration, type of exposure), existing studies in the literature are difficult to compare as they may not fully capture this variability. Furthermore, although NCs-based hydrogels offer better mechanical properties than conventional hydrogels, they have drawbacks in terms of strength and elasticity. Improving the mechanical characteristics of these materials is critical for their use in load-bearing tissues such as bone and cartilage. Additionally, they have a high-water content, which affects their swelling behavior. The degree of swelling can have an impact on the hydrogel’s efficacy in some applications, such as drug delivery and wound healing. Thus, controlling the swelling behavior is therefore critical. Another essential aspect is the need to develop more environmentally friendly, commercially viable and reproducible nanocellulose surface modification and processing procedures. A balance between novel functionalities and inherent properties of NCs-based materials needs to be found by tuning morphologies and surface properties. In parallel, synthesis and production methods for NCs assemblies should be further developed through scalable and commercially competitive manufacturing.

To conclude, additional research is required to fill existing gaps through the practical transfer from laboratory scale to commercial production, as well as to establish the practicality of the final products prior to introducing them to the market. Regardless of the difficulties outlined above, we anticipate that these ever-evolving innovative materials will be of great research interest in the biomedical field.

## 6. Conclusions

Many research studies have focused on obtaining the three types of NCs, especially for their potential to shape into high-performance NCs-based materials with tunable mechanical, physical and biological properties that can be used in biomedical fields. An overview of the number of scientific publications on NCs published over the last ten years indicates their significant increase, eclipsed by the explosive growth of publications on this subject recorded in the last three years. More than 50% of the studies on biomedical applications of NCs have been published in the last three years, a fact that underlines the increased interest in these unique materials.

This review highlights the great potential of NCs in its three different forms (CNCs, CNFs and BNC, respectively) and in three different medical fields: tissue engineering (TE), drug delivery (DD) and wound dressings (WD). The great structural versatility of NCs and the involvement of different concepts and pathways to construct materials with complex architectures and tunable properties is an essential advantage in their use in these three particular medical fields. The control of the crystallinity of NCs, their size and morphology, as well as the surface chemistry of NCs depends on the selection of appropriate cellulose resources and processing conditions. Thus, a good understanding of the properties of the individual NCs enables their further processing into different engineered structures (i.e., films, membranes, hydrogels, aerogels) with improved performance. For instance, NCs have proven to be a promising component in a variety of formulations for TE applications, demonstrating outstanding characteristics that can be applied in cartilage or bone tissue engineering or the forming of complex constructs with interconnected macropores and vascular-like structures, or they can be successfully used as bioink in 3D printing due to their excellent viscoelastic properties. Furthermore, NCs seem to be the ideal choice in advanced wound management strategies, for example in the encapsulation of antimicrobial agents for bacteria-associated wounds or as reinforcement material in the design of intelligent dressings with sensors that respond to specific changes in the wound environment and treating specific pathological issues at the molecular level.

Unquestionably, NCs have remarkable potential for the development of a new generation of biomedical materials, and this review thoroughly demonstrates the significant advancements that have been made in the last few years in the use of NCs-based hydrogels in a variety of medicine areas. Although further research is needed to fully understand their potential, this review attempts to gather high-quality information in order to represent a reference point for the scientific community in the effort to develop new NCs-based materials and to inspire their use in new biomedical fields of major interest.

## Figures and Tables

**Table 1:** Sources, preparation techniques and special characteristics of NCs.

Type	Sources	Preparation Methods	Special Characteristics	References
CNCs	Wood, cotton, hemp, wheat straw, tunicin, algae, bacteria	Acid hydrolysis	Short, rigid nanocrystals; woods (W/L): 5–40 nm/100–300 nm; non-woods (W/L): 7–25 nm/84–800 nm (cotton, wheat); 12–21 nm/107–215 nm (ramie); 25–30 nm/300–400 nm (BNC)	[12,13,14,15,16,17,18]
CNFs	Wood, sugar, beet, potato tuber, hemp, flax	Delamination before/after chemical or enzymatic treatment	Long, flexible nanofibers with significant amorphous content; web-like structures; W/L: 20–100 nm/several µm.	[11,19,20,21,22]
BNC	Low-molecular-weight sugars and alcohols	Bacterial synthesis	Highly crystalline 3D network, high purity; aggregated into nanofibrillar bundles; W = 50–150 nm; Static fermentation: uniaxially oriented ribbons; agitated fermentation: disordered, overlapping ribbon-like morphology.	[20,23,24,25]

Abbreviations: W—width; L—length.

**Table 3:** CNFs sources, preparation techniques and conditions.

Source	Preparation Technique	Preparation Conditions	References
Bagasse	a. Enzyme pretreatment b. Mechanical grinding	a. Novozymes endoglucanase/50 °C/12 h b. Ultrafine grinder, 10–15 J/1500 rpm	[56]
Cassava roots	a. Alkaline treatment b. Acid hydrolysis	a. 5% KOH/25 °C/14 h b. 30% SA/90 min/60 °C	[67]
	a. Alkaline treatment b. TEMPO-oxidation	a. 5% KOH/25 °C/14 h b. TEMPO/NaBr/NaClO	
Waste hemp	a. Alkali/bleaching treatment b. Acid hydrolysis	a. 2 wt/V% NaOH/50 °C/3 h; NaClO b. 64% wt/wt SA/45 °C/30 min	[68]
	a. Alkali/bleaching treatment b. Acid hydrolysis c. Ultrasonication	a. 4 wt% NaOH/80 °C/2 h; 1.7 wt% NaClO_2/_ABS (pH 4.8)/1 h/100 °C; b. 45%, 64% SA, FA, MA/60, 90 min/45°, 65 °C; c. 4 min/low speed.	[57]
Kenaf	a. Formic acid/acetic acid; b. Peroxyformic acid/ peroxyacetic acid c. Bleaching treatment d. Ball milling	a. 85% FA/AA/110 °C/2 h; b. 35% H_2_O_2_ with 85% FA/AA/80 °C/2 h; c. 35% H_2_O_2_/NaOH/80 °C/2 h; d. 30, 60, 90, 120 min.	[71]
Wheat straw			
Carrots residue	a. Blanching b. Refining c. Homogenization	a. 80 °C/1 h; b. PFI mill to 10,000 revolutions; c. Homogenizer: 2 wt%/5 passes/1000 bar.	[72]
Sugar beet	a. Steam Explosion b. Bleaching c. Ultrasonication	a. 220 °C/35 min/2.4 MPa; b. 6 wt% H_2_O_2/_80 °C/24 h; c. Ice/water bath/30 min/1000 W.	[73]

Abbreviations: SA—Sulfuric acid; FA—Formic acid; AA—acetic acid; MA—Maleic acid; PFA—Peroxyformic acid; PAA—peroxyacetic acid; TEMPO—2,2,6,6-tetramethylpiperidin-1-oxyl; ABS—acetate buffer solution.

**Table 4:** Optimization of BNC production using different bacteria strains, fermentation techniques and carbon sources.

Bacteria Strain	Fermentation Technique	Carbon Source	Optimal Fermentation Conditions	Productivity, g/L/day	Ref.
*G. xylinus* ATCC 700178	Static	Carob/Haricot bean	2.5 g/L carbon, T = 30 °C, pH = 5.5, t = 9 days	0.19	[83]
*G. xylinus* KCCM 41431	Static	Residual crude glycerol	20 g/L glycerol, pH = 5, t = 7 days	0.99	[87]
*G. xylinus* PTCC 1734	Static	Beet molasses/Cheese whey	T = 28 °C, pH = 5.5, t = 14 days	0.32	[18]
*G. sucrofermentans* B-11267	Dynamic	Wheat vinasse /Cheese whey	T = 28 °C, pH = 3.95–4.96, t = 3 days, 250 rpm	2.06	[86]
*A. xylinum* ATCC 23767	Static	Waste extract tobacco	T = 30 °C, pH = 6.5, t = 7 days	0.32	[88]
	Dynamic		T = 30 °C, 150 rpm	0.74	
*K. europaeus* SGP37	Static batch/Static intermittent fed-batch	Sweet lime pulp waste	T = 30 °C, pH = 6, t = 16 days; Addition every 48 h and 96 h	0.40	[89]
*K. xylinus* PTCC 1734	Static	Vinasse	40% vinasse, T = 30 °C, pH = 6, t = 10 days	0.18	[90]
*K. xylinus* PTCC 1734	Dynamic	Date syrup/Cheese whey	Date syrup: cheese whey ratio = 50:50, T = 28 °C, pH = 4.48, t = 10 days	0.19	[85]

Abbreviations: *G. xylinus—Gluconacetobacter xylinus*; *A. xylinum—Acetobacter xylinum*; *K. xylinus—Komagatacibacter xylinus*; *K. europaeus—Komagataeibacter europaeus*.

**Table 5:** Investigation techniques used to characterize the properties of NCs-based materials.

Scope	Investigation Technique	Properties
Structural	X-ray diffraction (XRD)	degree of crystallinity, type of allomorphs, interplanar distances, nanocrystallites size
	Solid-state cross-polarization magic angle spinning ^13^C NMR spectroscopy (CP/MAS ^13^CNMR)	degree of crystallinity, nanocrystallites lateral size, type of allomorph, degree of substitution
	Fourier transform infrared spectroscopy (FTIR)	functional groups, structural characteristics
	Raman spectroscopy (RS)	crystallinity
Morphological	Transmission electron microscopy (TEM)	morphology and dimensions (diameter, length, mean aspect ratio)
	Scanning electron microscopy (SEM)	
	Field emission-SEM (FE-SEM)	widths, length
	Transmission electron cryo-microscopy (Cryo-TEM)	structure of molecular aggregates
	Laser light scattering (LLS)	particle size, cross-sectional area
	Dynamic light scattering (DLS)	particle size, particle size distribution
	Zeta potential (ZP)	surface charge
Surface topography	Atomic force microscopy (AFM)	topography of the surface, diameter, length
	X-ray photoelectron spectroscopy (XPS)	chemical composition, binding constants, oxidation states
Elemental analysis	Gas chromatography (GC)	quantify individual chemical components in mixture; chemical purity
Chemical	Chemical methods	purity; content of functional groups
Mechanical	Dynamic mechanical analysis (DMA)	viscoelastic properties (storage modulus, loss modulus, damping parameter)
	Tensile testing	tensile strength; elongation at break
Rheological	Rheometry	shear stress, shear rate viscosity, thixotropy, viscoelastic properties
Biological	Biochemical methods	biodegradability
Thermal analysis	Thermogravimetric analysis (TGA), derivative thermogravimetry (DTG), differential thermal analysis (DTA), differential scanning calorimetry (DSC)	thermal stability, sorption/desorption processes, oxidation, thermal decomposition, thermo-physical characteristics

**Table 6:** An overview of the recent literature on NCs-based hydrogels for wound dressings.

Loaded Bioactive Agents	Scaffolds Involved	Biocompatibility Studies	Outcomes	Ref.
Antibiotics				
Levofloxacin	CNFs/CMC/DOP hydrogel composites	In vivo (burn wound model)	Accelerated wound healing by decreasing IL-1β expression	[146]
Bacitracin/ Amoxicillin	BNC gel membranes	-	Effective against *S. aureus* and *E. coli*	[147]
Vancomycin/ Ciprofloxacin	BNC grafted with GMA/BNC cross-linked with EGDMA	-	Effective against *S. aureus* and *Klebsiella pneumoniae*	[148]
Neomycin	PVA/B gel reinforced with OCMC-DA and CNFs	In vitro (NIH3T3 fibroblasts)	Effective against *E. coli* and *S. aureus*	[162]
Clindamycin hydrochloride	CNFs/LMP/SA hydrogel	In vitro (HaCaT cell)	Good biocompatibility; increased cell viability	[150]
Silver nanoparticles				
	CNFs/sacran hydrogel	-	Effective against *E. coli*, *S. aureus* and *P. aeruginosa*	[151,152]
	BNC hydrogel	-	Effective against *S. aureus*, *P. aeruginosa* and *C. auris*; Antioxidant properties	[163]
	CNFs/GEL hydrogel	In vivo (full-thickness rat cutaneous wound model)	Effective against *E.coli*; improved wound healing ability	[164]
Metal cations				
Manganese, cobalt, copper, zinc, silver, and cerium	BNC/ALG double-network hydrogels	In vitro (L929 fibroblast cells); In vivo (rat cutaneous wound model)	Effective against *E. coli* and *S. aureus*; improved wound healing ability	[153]
Natural compounds				
Thymol	BNC hydrogel	In vitro (NIH 3T3 fibroblasts); in vivo (Wistar rats)	Effective against *E. coli* and *S. aureus*, *P.aeruginosa* and *Klebsiella pneumoniae*; increased cell viability and acceleration in wound repair	[154]
Alpha- tocopherol	TEMPO-oxidized CNFs/ALG/P	In vitro (L929 fibroblast cells); in vivo (diabetic rat model)	Effective healing of full-thickness skin wounds in diabetic rats	[155]
Propolis	BNC hydrogels	-	Effective against *S. aureus*	[156]
Curcumin	BNC hydrogels	In vitro (human dermal fibroblasts)	Effective against *S. epidermidis* and *E. coli.*	[157]
Oregano essential oil	CNFs/GEL/PVP composite hydrogel	In vitro (NIH/3 T3 fibroblasts); in vivo (diabetic rat model)	Increased granulation, enhanced re-epithelialization, and a drastic decrease in inflammation in diabetic rat models with foot ulcers	[158]
Vitamin C and E	BNC/PUL bilayer wound dressings	In vitro (L929 fibroblasts)	Antioxidant properties; accelerated wound closure and collagen synthesis	[159]
Resveratrol	PVA/B/Resveratrol grafted CNFs	In vitro (L929 cells); in vivo (female KM mice)	Antioxidant properties; Effective against *S. aureus*; enhanced wound closure capabilities	[161]
Lawsone	TEMPO-oxidized CNFs/CHI sponges	In vitro (L929 fibroblast cells); in vivo (male Sprague-Dawley rats)	Hemostatic feature and accelerated cutaneous wound healing in rat punch biopsy model	[165]

Abbreviations: CMC—carboxymethylcellulose; DOP—dopamine; GMA—glycidylmethacrylate; EGDMA—ethylene glycol dimethacrylate; PVA—poly(vinyl alcohol); OCMC-DA—dopamine-grafted oxidized carboxymethyl cellulose; LMP—low methoxy pectin; SA—sodium alginate; HaCaT cell—human epidermal keratinocytes; GEL—gelatin; ALG—alginate; P—pluronic^®^ F-127; PVP—polyvinyl pyrollidone; PUL—pullulan; B—borax; CHI—chitosan; *S. aureus*—*Staphylococcus aureus*; *E. Coli*—*Escherichia coli*; *P. aeruginosa*—*Pseudomonas aeruginosa*; *C. auris*—*Candida auris*; *S. epidermidis*—*Staphylococcus epidermidis.*

**Table 7:** An overview of the recent literature on NCs-based hydrogels for TE applications.

Cellulose Type	Materials/Scaffolds Involved	Biocompatibility Studies	TE Application	Ref.
CNFs	CNFs-GEL crosslinked hydrogels	In vitro (hBMSCs)	Bone	[180]
	CNFs-PEGDMA injectable hydrogels crosslinked with Irgacure 2959	In vitro injection model	Nucleus pulposus	[181]
	CNFs-CHI composite hydrogels	In vitro (fibroblast cells); ex vivo (spine pig models)	Nucleus pulposus	[182]
	CNFs-CHI self-healing hydrogels	In vitro (NSCs)	Neural regeneration	[183]
	CNFs-MC-CMC-PEG thermo-responsive injectable hydrogels	In vitro (RBMSCs and L929 fibroblast cells); in vivo (wall-cecal abrasion model in rats)	Post-surgical peritoneal tissue adhesion	[184]
	CNFs–SF-PEEK injectable composite hydrogels	In vitro(hDPSCs); in vivo (rats)	Bone (craniofacial region)	[185]
CNCs	CNCs-CHI-Pectin injectable hydrogels	In vitro (chondrocytes)	Cartilage	[186]
	CNCs-CHI-MTA injectable hydrogels	In vitro (hDPSCs)	Tooth (dentin- pulp complex)	[187]
	CNCs-Fbg injectable composite hydrogels	In vitro (mouse myoblasts)	Muscle (myotube formation)	[188]
	CNCs-PVA composite hydrogels	In vitro (HCE-2 cells)	Cornea regeneration	[189]
	CNCs-MeGel photocrosslinkable composite hydrogels	In vitro (HADMSC)	Cardiac tissue	[190]
	Modified CNCs-CHI-Ag injectable hydrogels	In vitro (HUVECs); in vivo (mice)	Skin	[191]
BNC	BNC-PAA-GO composite hydrogels	In vitro (hDF)	Skin	[192]
	BNC-MeGel photocrosslinkable composite hydrogels	In vitro (HC-a)	Cartilage	[193]
	BNC-GNPs composite hydrogels	In vitro (hBMSCs); in vivo (rabbits)	Bone	[194]
	BNC-PVA composite hydrogels	In vitro (hCSCs)	Corneal stroma	[195]
	BNC-GEL-SeNPs composite hydrogels	In vivo (rats)	Skin	[196]
	BNC-LAP in-situ nanocomposites	In vitro (HaCat cells)	Skin	[197]

Abbreviations: GEL—gelatin; PEGDMA—poly(ethylene glycol) dimethacrylate; hBMSCs—human bone marrow mesenchymal stem cells; CHI—chitosan; MC—methyl cellulose; CMC—carboxymethyl cellulose; PEG—polyethylene glycol; SF—silk fibroin; PEEK—polyetheretherketone; NSCs—neural stem cells; RBMSCs—bone marrow mesenchymal stem cells; hDPSCs—human dental pulp stem cells; MTA—mineral trioxide aggregate; Fbg—fibrinogen; PVA—poly(vinyl alcohol); HCE-2 cells—human corneal epithelial cells; MeGel-methacrylated gelatin; HADMSC—human adipose-derived mesenchymal stem cells; HUVECs—human umbilical vein endothelial cells; PAA—poly(acrylic acid); GO—graphene oxide; hDF—human dermal fibroblast; HC-a—human articular chondrocytes); GNPs—gold nanoparticles; hCSCs—human corneal stromal cells; SENPs—selenium nanoparticles; LAP—Laponite; HaCat cells—human immortalized keratinocytes.

**Table 8:** The most recent hydrogels based on NCs for DD, according to the administration route.

Administration Route	Materials Involved	Drug	Ref.
Oral	BNC hydrogels loaded with drug-β-CD inclusion complex	Berberine hydrochloride	[225]
	CNFs/SA interpenetrating network hydrogels	Aspirin	[226]
	Su-CNCs/PNIPAm pH and thermo-responsive hydrogels	Famotidine	[227]
	CNFs/SA pH-responsive hydrogels	Ibuprofen	[228]
	Magnetic Fe_3_O_4_ NPs/CNCs/PNIPAm nanocomposite hydrogels	Vancomycin	[229]
	CNCs/CHI composite hydrogel cylinders	Bovine serum albumin	[230]
Ocular	PAA grafted CNCs mucoadhesive hydrogels	Cisplatin	[231]
Transdermal	BNC gel membranes	Caffeine, Lidocaine, Ibuprofen, Diclofenac	[232]
Subcutaneous	3D printed PLA non-active capsules filled with anionic CNFs hydrogel	Beta blockers metoprolol and nadolol	[233]
Intratumoral	CNCs/AG loaded with PDA fluorescent injectable hydrogel	Paclitaxel	[234]
	Injectable temperature, pH, and NIR tri-stimuli-responsive composite hydrogel based on CNFs and citric-acid-stabilized Prussian blue nanoparticles	Doxorubicin	[235]
	ALA/Fe/CNCs or PDA/Fe/CNCs pH-responsive nanohydrogels	Paclitaxel	[236]
	PDA/CNCs/PNIAPm nanocomposite hydrogels	5-Fluorouracil	[237]
Topical	MPDA@GO/CNFs composite hydrogel	Tetracycline hydrochloride	[238]
	CNFs loaded hydrogel with CO nanoparticles	Surfactin and Herbmedotcin	[239]
Local	Sulfonated CNFs/GEL hydrogels	Antigen (ovalbumin)	[240]
	NCs-GEL hydrogels	Astragaloside IV	[241]

Abbreviations: β-CD—β-cyclodextrin; SA—sodium alginate; Su-CNCs—succinylated cellulose nanocrystals; PNIPAm—poly(N-isopropylacrylamide); Fe_3_O_4_ NPs—Fe_3_O_4_ nanoparticles; PLA—poly(lactic acid); PAA—poly(acrylic acid); CHI—chitosan; PAA—poly(acrylic acid); AG—agarose; PDA—polydopamine; ALA—5-aminolevulinic acid; MPDA—mesoporous polydopamine; GEL—gelatin; CO—κ-carrageenan oligosaccharides.

## Data Availability

Not applicable.
